# STAT1 Hyperphosphorylation and Defective IL12R/IL23R Signaling Underlie Defective Immunity in Autosomal Dominant Chronic Mucocutaneous Candidiasis

**DOI:** 10.1371/journal.pone.0029248

**Published:** 2011-12-14

**Authors:** Sanne P. Smeekens, Theo S. Plantinga, Frank L. van de Veerdonk, Bas Heinhuis, Alexander Hoischen, Leo A. B. Joosten, Peter D. Arkwright, Andrew Gennery, Bart Jan Kullberg, Joris A. Veltman, Desa Lilic, Jos W. M. van der Meer, Mihai G. Netea

**Affiliations:** 1 Department of Medicine, Radboud University Nijmegen Medical Centre, Nijmegen, The Netherlands; 2 Department of Human Genetics, Radboud University Nijmegen Medical Centre, Nijmegen, The Netherlands; 3 Department of Rheumatology, Radboud University Nijmegen Medical Centre, Nijmegen, The Netherlands; 4 Nijmegen Institute for Infection, Inflammation, and Immunity (N4i), Nijmegen, The Netherlands; 5 Royal Manchester Hospital, University of Manchester, Manchester, United Kingdom; 6 Institute for Cellular Medicine, Newcastle University, Newcastle upon Tyne, United Kingdom; University Freiburg, Germany

## Abstract

We recently reported the genetic cause of autosomal dominant chronic mucocutaneous candidiasis (AD-CMC) as a mutation in the *STAT1* gene. In the present study we show that *STAT1* Arg274Trp mutations in the coiled-coil (CC) domain is the genetic cause of AD-CMC in three families of patients. Cloning and transfection experiments demonstrate that mutated *STAT1* inhibits IL12R/IL-23R signaling, with hyperphosphorylation of STAT1 as the likely underlying molecular mechanism. Inhibition of signaling through the receptors for IL-12 and IL-23 leads to strongly diminished Th1/Th17 responses and hence to increased susceptibility to fungal infections. The challenge for the future is to translate this knowledge into novel strategies for the treatment of this severe immunodeficiency.

## Introduction

Chronic mucocutaneous candidiasis (CMC) is a hereditary primary immunodeficiency characterized by severe skin and mucosal *Candida* infections, dermatophytosis and onychomycosis [1]. CMC is a heterogeneous syndrome, the best characterized clinical entities being the autosomal recessive autoimmune polyendocrinopathy candidiasis ectodermal dystrophy (APECED) syndrome and autosomal dominant CMC (AD-CMC) [2].

APECED is due to mutations in the gene *autoimmune regulator* (AIRE) [3], and these explain the autoimmune phenomena, including autoantibodies against the antifungal cytokines interleukin (IL)-17A, IL-17F and IL-22 [4], [5]. In contrast, the genetic cause of AD-CMC has remained unknown until very recently. In a study performed in 5 families with AD-CMC, we reported that the disease is caused by mutations in the gene coding for the Signal Transducer and Activator of Transcription (STAT)1 signaling molecule [6]. The discovery of *STAT1* mutations as cause of AD-CMC was remarkable, as STAT1 deficiency had been previously reported to be associated with mycobacterial and viral, but not fungal, infections [7], [8]. The presence of the AD-CMC mutations in the coiled-coil (CC) domain of *STAT1*, rather than in the Src homology 2 (SH2) or DNA-binding domains of the protein as in patients with mycobacterial/viral infections, is believed to explain the difference [6]. Nevertheless, the cellular and molecular mechanisms responsible for the increased susceptibility to fungal infections in patients with AD-CMC and *STAT1* mutations remain to be deciphered.

In the present study we assessed the presence and function of *STAT1* mutations in the index family published previously [6], and in two additional previously unreported families with AD-CMC. We also assessed the immune abnormalities underlying the increased susceptibility to infections in these patients.

## Results and Discussion

In the present study we report that mutations in the CC-domain of STAT1, the genetic cause of AD-CMC, lead to hyperphosphorylation of STAT1 resulting in increased responsiveness to IFN-γ and impaired IL-12 and IL-23 signaling pathways. We strengthened our recent observation that STAT1 mutations are responsible for AD-CMC [6] by showing the Arg274Trp mutation in the CC-domain of STAT1 in patients from three additional families with AD-CMC. These data further establish the role of STAT1-mediated signaling as a crucial mechanism for mucosal antifungal defense.

### Missense mutation in *STAT1*

**Figure 1:**
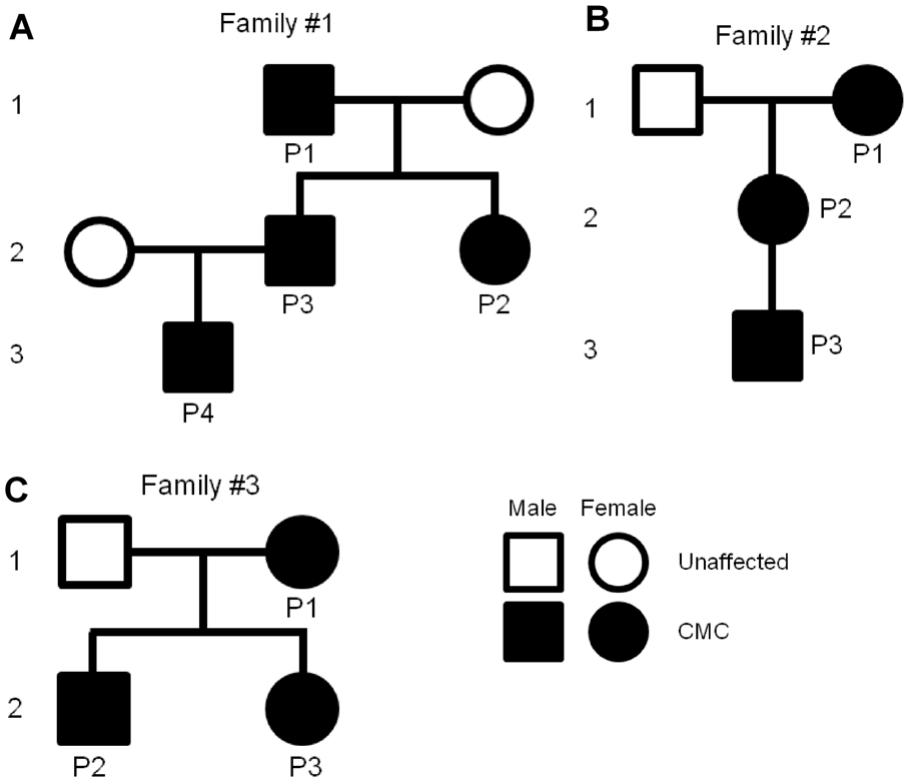
Pedigrees of three families with AD-CMC. (A) Pedigree of a Dutch family with four patients affected from 3 generations. (B) Pedigree of a UK sample with 3 patients affected from 3 generations. (C) Pedigree of a UK sample with 3 patients affected from 2 generations. Patients with candidiasis  =  closed black symbols; male patients  =  squares; female patients  =  circles.

Individual P4 from the Dutch family (#1) (Figure 1A) was demonstrated to carry the Arg274Trp mutation in exon 10 of STAT1 similar to his father (P3), aunt (P2) and grandfather (P1) [6]. The same *STAT1* variant (c.820C>T; p.Arg274Trp) was detected in all three individuals from the British family #2 (Figure 1B), and all three individuals from British family #3 (Figure 1C) (Figure 1D). The discovery of *STAT1* mutations as a cause of AD-CMC was rather surprising, as mutations in the SH2 or DNA-binding domains of the protein had been previously shown to be associated with mycobacterial and viral, but not fungal, infections [9], [10]. We therefore sought to identify the molecular mechanism behind this difference.

### Lower production of IFN-γ and IL-17 in AD-CMC patients

**Figure 2:**
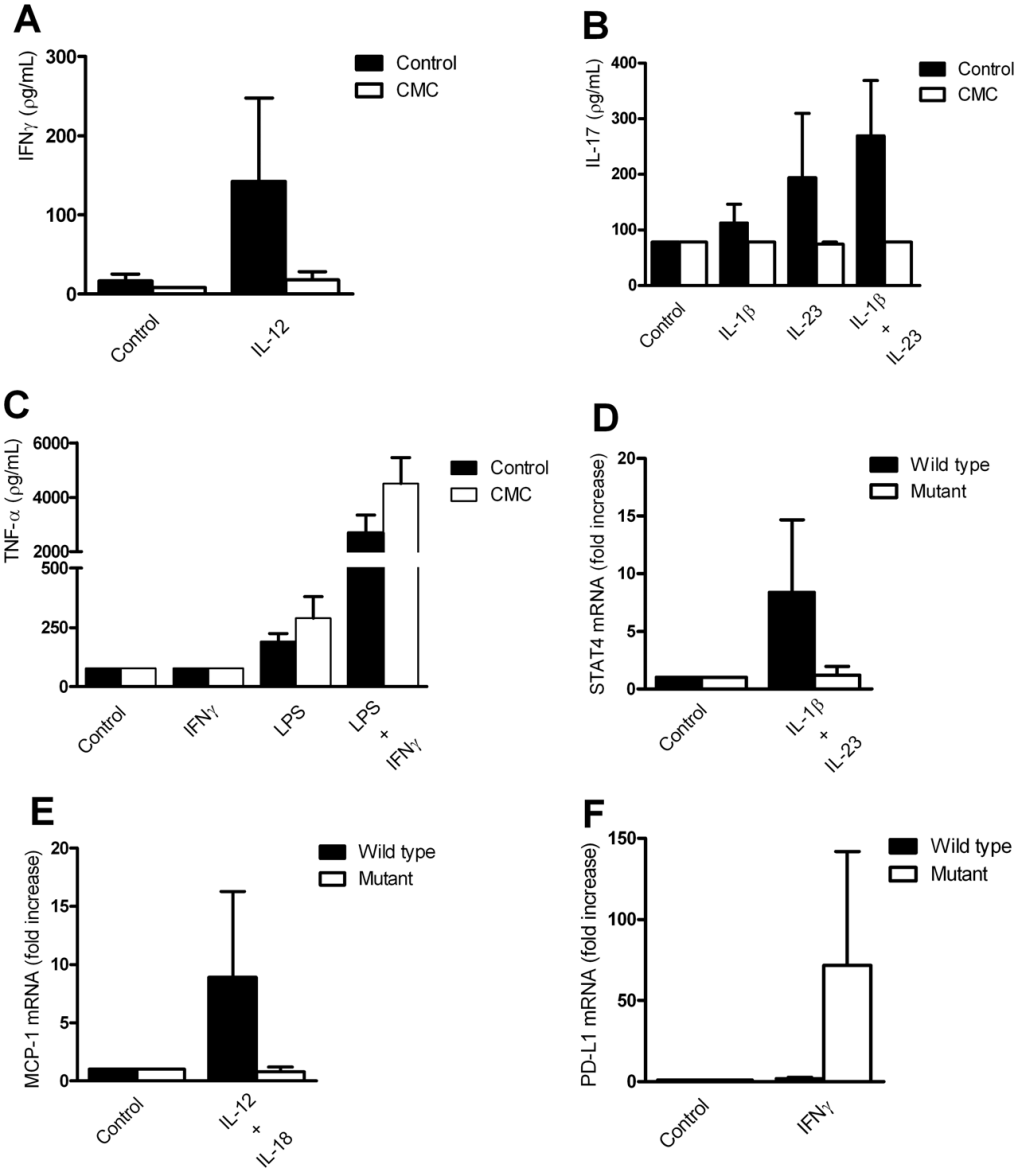
AD-CMC cells or transfection of cells with the mutated STAT1 inhibited IL-12R- and IL-23R-induced genes, but increased IFN-γ signaling. Defective IL-12R (A) and IL-23R (B) pathways in cells isolated from AD-CMC patients. Data represent mean + SEM from 3 healthy controls and 3 AD-CMC patients (P3 and P4 from family #1 and P3 from family 2). (C) Stimulation of the IFN-γ receptor pathway results in increased production of the proinflammatory cytokine TNFα. Data represent mean + SEM from 3 healthy controls and 3 AD-CMC patients. CD4^+^ T cells from healthy controls were transfected with wild-type or mutant STAT1 plasmid. (D) Decreased transcription of STAT4 upon stimulation with IL-1β (10 ng/mL) and IL-23 (50 ng/mL), (E) and decreased transcription of MCP1 upon IL-12 (10 ng/mL) and IL-18 (10 ng/mL) stimulation. (F) The CD4^+^ cells transfected with mutant STAT1 plasmid showed increased transcription of PD-L1 upon stimulation with IFN-γ (1 µg/mL). Data represent mean + SEM from 6 healthy controls from 3 separate experiments.

IL-12 induced no IFN-γ production in cells obtained from AD-CMC patients (Figure 2A), and IL-1β and IL-23 induced less IL-17 production in cells from P3 and P4 from family #1 (Figure 2B). In contrast, production of TNF-α in response to IFN-γ and LPS was higher in PBMCs from CMC patients compared to healthy controls (Figure 2C). With these findings we have confirmed our observations in experiments with cells from other AD-CMC patients, as reported previously [6].

### Arg274Trp *STAT1* inhibits signaling via IL-12R and IL-23R

The transcription of several IFN-γ-regulated genes was measured in CD4^+^ T-cells from healthy controls transfected with either wild-type *STAT1* plasmid or mutant *STAT1* plasmid. The cells were stimulated for 24 hours with either IFN-γ (1 µg/mL), or IL-β (10 ng/mL) and IL-23 (50 ng/mL), or IL-12 (10 ng/ml) and IL-18 (10 ng/ml). In line with the experiments performed in primary cells isolated from AD-CMC patients, CD4^+^ T-cells transfected with the mutated *STAT1* allele, but not with the wild-type allele, exhibited decreased transcription of *STAT4* after exposure to IL-1β and IL-23 (Figure 2D), and decreased transcription of *MCP1* upon IL-12 and IL-18 stimulation (Figure 2E). In contrast, CD4^+^ T-cells transfected with the mutant *STAT1* plasmid showed increased transcription of PD-L1 upon stimulation with IFN-γ (Figure 2F). Cells that were transfected with STAT1 plasmid exhibited highly increased expression of STAT1 as compared to untransfected cells. Furthermore, no difference in STAT1 mRNA expression was observed between cells either transfected with wild-type or mutant STAT1 plasmid (Figure S1). These data correct for the possibility that the observed effects are due to a differential expression of the wild-type compared to the mutant STAT1 plasmid.

Based on the results of these transfection studies, it can be concluded that the differences in gene expression observed in AD-CMC patients as compared to healthy controls can be fully ascribed to the presence of the Arg274Trp mutation in STAT1.

### Increased phosphorylation and activity of STAT1 in AD-CMC patients

**Figure 3:**
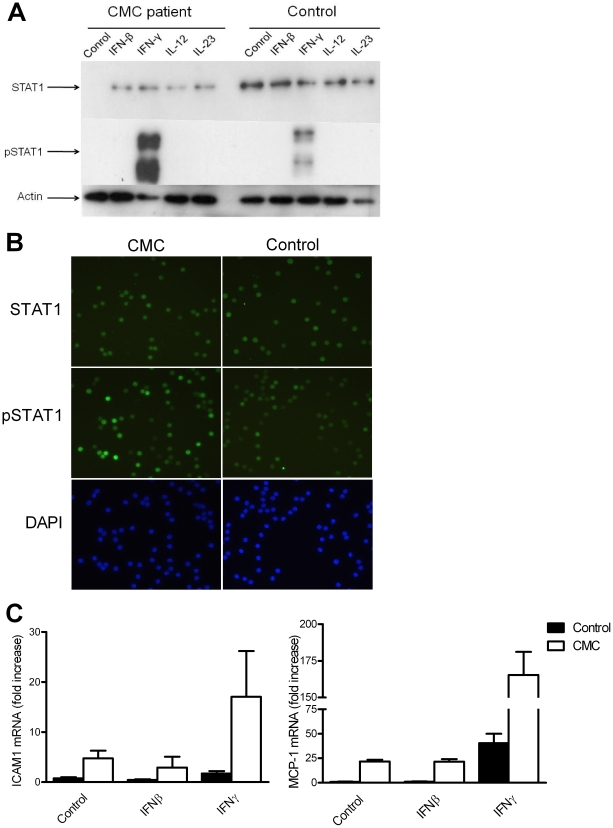
Increased STAT1 phosphorylation in cells of AD-CMC patients. (A) PBMC from two healthy controls and two AD-CMC patients were stimulated for two hours with culture medium, IFN-β (500 U/mL), IFN-γ (1 µg/mL), IL-23 (50 ng/mL) or IL-12 (10 ng/mL). Cells were lysed, and total STAT1, phosphorylated STAT1 and actin were assessed by Western blot. Figure is representative of two separate experiments. (B) Increased immunofluorescence of pSTAT1 in IFN-γ-stimulated CD4^+^ cells from AD-CMC patients. Figure is representative of three separate experiments. (C) PBMCs from three controls and two AD-CMC patients were stimulated with culture medium, IFN-β, or IFN-γ for two hours. The transcription of ICAM1 and MCP-1 was measured using RT-PCR. Bars represent means + SEM.

The consistent finding of increased responsiveness to IFN-γ in cells from patients and in cells transfected with the mutated STAT1 suggested a gain of function of STAT1. We therefore assessed whether the Arg274Trp mutation affects phosphorylation of STAT1. PBMCs were incubated with various stimuli and STAT1 phosphorylation was assessed by Western blotting. Phosphorylation of STAT1 after IFN-γ stimulation was higher in AD-CMC patients than in healthy controls (Figure 3A). The lower bands observed on the Western blot of phosphorylated STAT1 very likely represent breakdown products of the phosphorylated STAT1 protein. Immunofluorescence microscopy also demonstrated increased phosphorylation of STAT1 in IFN-γ-stimulated CD4^+^ cells from AD-CMC patients, but no difference in total STAT1 (Figure 3B). These data are supported by Begitt et al, demonstrating that mutations in the CC domain of STAT1 result in hyperphosphorylation and prolonged entrapment of STAT1 in the nucleus, leading to inhibited nuclear export of STAT1 [11]. Also, Lui et al. demonstrated that the Arg274Trp mutation in STAT1 leads to increased phosphorylation of STAT1 [12].

Upon IFN-γ stimulation through the IFN-γ receptor in physiological conditions, the downstream signaling molecules JAK1, JAK2 and STAT1 are activated [13]. Upon activation, STAT1 forms homodimers, which translocate to the nucleus, in order to induce transcription of numerous genes, including ICAM-1[14] and MCP-1 [15]. We measured the transcription of several IFN-γ regulated genes in AD-CMC patients and in healthy controls, to determine whether the Arg274Trp mutation in *STAT1* affects its function as a transcription factor. When PBMCs were stimulated for two hours with IFN-γ (1 µg/mL), AD-CMC patients showed increased transcription of ICAM1 and MCP-1 compared to healthy controls (Figure 3C). The transcription of STAT1, STAT3, IDO and PD-L1 was also increased (data not shown), supporting a gain of function mechanism for STAT1 in AD-CMC. Moreover, also unstimulated cells from AD-CMC patients exhibited elevated expression of these genes compared to cells from healthy controls.

How do these molecular mechanisms explain the defective antifungal defense in AD-CMC patients? In the present study we show that these patients display significant deficiencies in the capacity to mount Th1 and Th17 responses, due to defective IL-12R and IL-23R pathways. The contributing role of a defective IL-12R signaling for CMC is strengthened by the observation that 23% of patients with isolated IL-12R deficiency have a mild form of CMC [16]. However, STAT1 mutations in the CC domain additionally lead to a defective response to the cytokines IL-1β and IL-23. These cytokines play an important role in the development of Th17 responses, which in turn are crucial in the host defense against mucosal fungal infections. This notion is derived from the observation that APECED patients develop neutralizing anti-IL-17 and anti-IL-22 antibodies during the course of the disease [4], and that hyper-IgE syndrome patients, who suffer from chronic onychomycosis and oropharyngeal candidiasis, display defective Th17 responses due to STAT3 defects [17]. In line with this, we and others have also reported chronic fungal infections in patients with defects in the dectin-1/CARD9 pathway, leading to defective Th17 responses [18], [19]. We have also previously reported decreased IL-17 production and Th-17 cell proliferation in patients with AD-CMC [20]. Defective Th17 responses, in turn, are accompanied by decreased production of IL-17 and IL-22 which translates into decreased production of antifungal beta-defensins as well as impaired influx of neutrophils [21], [22]. Therefore, the combined deficiency in IL-12R signaling and impaired Th17 responses are likely to explain the susceptibility to fungal infections in patients with AD-CMC.

In conclusion, we strengthen the very recent observations that *STAT1* mutations in the CC-domain are the genetic cause of AD-CMC. Moreover, we have now deciphered the cellular and molecular mechanisms leading to this clinical phenotype: hyperphosphorylation of STAT1, resulting in hyper-responsiveness to IFN-γ stimulation, but defective IL-12R and IL-23R pathways. In the end, these defects result in markedly diminished Th1/Th17 immunity leading to increased susceptibility to fungal infections. Future studies are warranted to investigate the disease mechanisms in more detail, especially to elucidate the extent of which STAT1-STAT3 and STAT1-STAT4 heterodimers are formed in AD-CMC patients upon stimulation with either IL-12 or IL-23. Furthermore, the challenge for the future will be to assess the proportion of patients with AD-CMC, but also sporadic CMC, that harbor STAT1 mutations, but especially to translate this knowledge into novel strategies for the treatment of this severe immunodeficiency.

## Materials and Methods

### Family #1

The clinical characteristics and pedigree of a Dutch Caucasian non-consanguinous family are represented in Figure 1A and in Table S1. The patients of the first two generations have been previously presented [6]. Since then, the son of one of the patients from the second generation was also diagnosed with the disease. The patients have severe chronic oropharyngeal candidiasis and dermatophytosis of the feet.

### Family #2

The clinical characteristics and pedigree of a non-consanguinous British Caucasian family are presented in Figure 1B and in Table S1. Three generations are affected with severe persistent candidiasis of the oropharynx, nails and skin (perineum in the child), all have iron-deficiency anemia. The grandmother and daughter have hypothyroidism while the boy has thyroid (peroxidise) autoantibodies but has still not developed clinical hypothyroidism.

### Family #3

The clinical characteristics and pedigree of a non-consanguinous British Caucasian family are presented in Figure 1C and Table S1. The mother, her son and daughter all suffer with oral candidiasis and hypothyroidism. The children unusually also suffer with mouth ulcers and herpetic whitlow.

### Ethics approval

The study was approved by the Ethics Committee of Radboud University Nijmegen Medical Centre, and the Newcastle and North Tyneside Local Research Ethics Committee. Informed consent was obtained from all family members and healthy controls.

### Sequencing of *STAT1* and *AIRE* mutations

To assess for the presence of *STAT1* mutations in the patients, conventional PCR and Sanger sequencing were performed. All coding exons of the CC-domain of *STAT1*, including exon 10, were amplified and analyzed. *AIRE* mutations were excluded by sequencing the gene as previously described [13].

### Cell isolation and stimulation

PBMCs were isolated by density gradient centrifugation of PBS-diluted blood (1∶1) over Ficoll-Paque, as previously described [6]. T cells were positively selected using CD4 microbeads (130-045-101, Miltenyi Biotec, Utrecht, the Netherlands). Cells (5×10^6^/ml) were stimulated in 96-well plates (Greiner, Nuremberg, Germany), with combinations of IFN-γ (1 µg/mL) (Boehringer Ingelheim, Alkmaar, the Netherlands) and LPS (1 ng/ml), IL-1β (10 ng/ml) and IL-23 (10 ng/mL), or IL-12 (1 ng/ml) and IL-18 (10 ng/ml). After 24 or 48 hours (without serum) or 5 days of incubation (in the presence of 10% serum), cytokine concentrations were measured using ELISA: TNF-α, IL-17, IL-22 (R&D Systems); IFN-γ (Pelikine).

### Western blotting

For immunoblotting, 10×10^6^ cells were lysed in 100 ml lysis buffer (50 mM Tris, 1 mM EDTA, 150 mM NaCL, 1% ND40, 5 mM NaF, 0.05% Sodium Deoxycholate, PhosSTOP and cOmplete proteinase inhibitor cocktail (Roche, Almere, the Netherlands)). The homogenate was frozen, thawed then centrifuged at 4 °C for 10 min at 14,000 rpm, and the supernatant was taken for Western blotting. Equal amounts of protein were subjected to SDS-PAGE using 10% and 15% polyacrylamide gels at a constant voltage of 100V. After SDS-PAGE, proteins were transferred to nitrocellulose membrane (0.2 mm). The membrane was blocked with 5% (w/v) milk powder in PBS for 1 hour at room temperature followed by incubation overnight at 4°C with a STAT1 or pSTAT1 (Tyr701) antibody (9172 and 9167 respectively, Cell Signaling, Leiden, the Netherlands) in 5% BSA/TBS/T (5% bovine serum albumin/Tris-buffered saline/Tween 20). After overnight incubation the blots were washed three times with TBS/T and incubated with HRP-conjugated goat anti-rabbit antibody at a dilution of 1∶10,000 in 5% (w/v) milk powder in PBS for 1 hour at room temperature. After washing the blots three times with TBS/T, the blots where developed with ECL according to manufacturer's instructions (GE Healthcare, Diegem, Belgium).

### STAT1 Immunofluorescence

CD4^+^ cells were stimulated for 10 minutes with IFN-γ on cover slides. Subsequently, the cells were fixed with 10% PFA, and stained with α-pSTAT1 (Tyr701) or α-STAT1 and goat anti-rabbit Alexa 488 according to manufacturer instructions. The coverslides were mounted using Vectashield containing DAPI.

### STAT1 cloning and transfection experiments

The Arg274Trp mutation was introduced into a plasmid containing the human STAT1α gene (pUNO-hSTAT1a, Invivogen, Toulouse, France), using the QuickChange^TM^ Site-Directed Mutagenesis Kit (200518, Stratagene, Eindhoven, the Netherlands). The following primers were used for the PCR reaction: 5′- GAG-AGT-CTG-CAG-CAA-GTT-TGG-CAG-CAG-CTT-AAA-AAG-3′ (forward) and 5′- CTT-TTT-AAG-CTG-CTG-CCA-AAC-TTG-CTG-CAG-ACT-CTC-3′ (reverse). Human CD4^+^ cells were transfected using HIPerfect reagent (Qiagen, Venlo, the Netherlands), according to manufacturer instructions.

### Quantitative PCR of IL-12R/IL-23R/IFN-γ-responsive genes

One million freshly isolated PBMCs or (transfected) CD4^+^ cells were stimulated with IFN-γ, IL-β and IL-23 or IL-12 and IL-18. After two hours, total RNA was extracted in 400 µL TRIzol reagent (Invitrogen). Isolated RNA was reverse transcribed into complementary DNA using oligo(dT) primers and MMLV reverse transcriptase. PCR was performed using a 7300 realtime PCR system (Applied Biosystems, Lennik, Belgium). Primer sequences are presented in Supplemental Information. PCR conditions were as follows: two minutes at 50°C and 10 minutes at 95°C, followed by 40 cycles of PCR reaction at 95°C for 15 seconds, and 60°C for one minute. Primer sequences used for RT-PCR assessment were: STAT4: 5′-ACA-ATG-AAA-CCA-TGG-CAA-CGA-3′ (forward) and 5′-TGA-AAT-TTT-CCC-TGA-AGG-ACC-TTC-3′ (reverse); ICAM: 5′-TTG-AAC-CCC-ACA-GTC-ACC-TAT-3′ (forward) and 5′-CCT-CTG-GCT-TCG-TCA-GAA-TC-3′ (reverse); MCP-1: 5′-CCA-GTC-ACC-TGC-TGT-TAT-AAC-3′ (forward) and 5′-TGG-AAT-CCT-GAA-CCC-ACT-TCT-3′ (reverse); PD-L1: 5′-GCT-GAA-CGC-ATT-TAC-TGT-CAC-G-3′ (forward) and 5′-AGT-GCA-GCC-AGG-TCT-AAT-TGT-3′ (reverse). B2M was used as a reference housekeeping gene, for which the primers were: 5′- ATG-AGT-ATG-CCT-GCC-GTG-TG-3′ (forward) and 5′- CCA-AAT-GCG-GCA-TCT-TCA-AAC-3′ (reverse).

## Supporting Information

Figure S1 **STAT1 expression in transfected CD4^+^ T-cells.** STAT1 mRNA expression in CD4^+^ T cells untransfected or transfected with wild-type or mutant STAT1 plasmid. Cells were left unstimulated or stimulated with IFN-γ for 1 hour.
(DOC)

Table S1 **Clinical characteristics of 8 patients with AD-CMC from 3 families.**
(DOC)
