# Supplementary material for: STAT1 Hyperphosphorylation and Defective IL12R/IL23R Signaling Underlie Defective Immunity in Autosomal Dominant Chronic Mucocutaneous Candidiasis
Source: PLoS One. 2011 Dec 14;6(12):e29248. doi: 10.1371/journal.pone.0029248 (PMC3237610; doi:10.1371/journal.pone.0029248)

**Supplementary information submitted along with the following manuscript:**

**STAT1 hyperphosphorylation and defective IL12R/IL23R signaling underlie defective immunity in autosomal dominant chronic mucocutaneous candidiasis**

Sanne P. Smeekens^1,4,*^, Theo S. Plantinga^1,4,*^, Frank L. van de Veerdonk^1,4,*^, Bas Heinhuis^3^, Alexander Hoischen^2^, Leo A.B. Joosten^1,4^, Peter Arkwright^5^, Andrew Gennery^6^, Bart Jan Kullberg^1,4^, Joris A. Veltman^2^, Desa Lilic^6^ , Jos W.M. van der Meer^1,4,#^, Mihai G. Netea^#1,4,@^

**Supplemental Figure 1. STAT1 expression in transfected CD4^+^ T-cells.**

STAT1 mRNA expression in CD4^+^ T cells untransfected or transfected with wild-type or mutant STAT1 plasmid. Cells were left unstimulated or stimulated with IFN-γ for 1 hour.


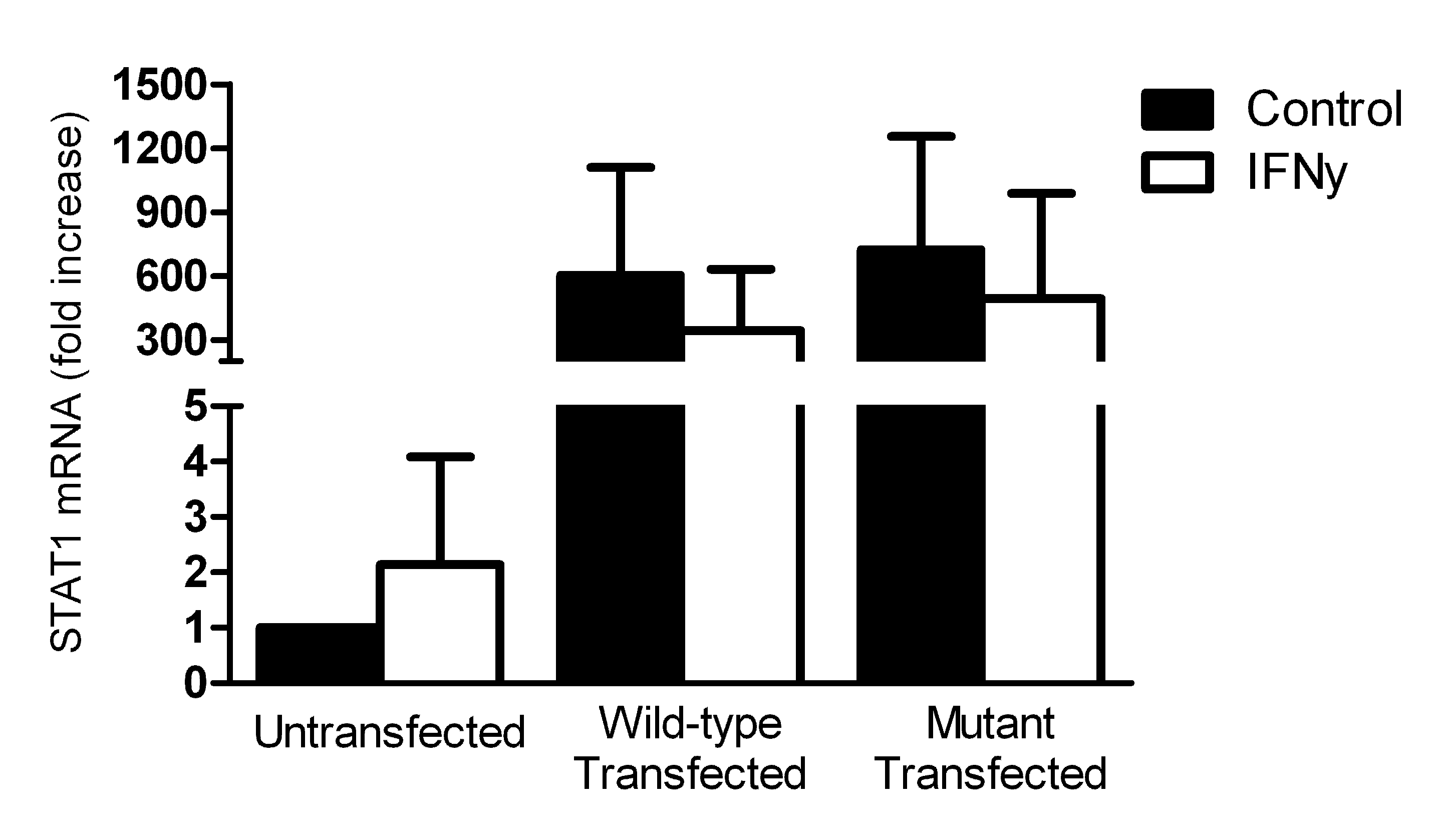

Supplement: Figure S1 — STAT1 expression in transfected CD4+ T-cells. STAT1 mRNA expression in CD4+ T cells untransfected or transfected with wild-type or mutant STAT1 plasmid. Cells were left unstimulated or stimulated with IFN-γ for 1 hour. (DOC) [file pone.0029248.s001.doc]
