# Supplementary material for: STAT1 Hyperphosphorylation and Defective IL12R/IL23R Signaling Underlie Defective Immunity in Autosomal Dominant Chronic Mucocutaneous Candidiasis
Source: PLoS One. 2011 Dec 14;6(12):e29248. doi: 10.1371/journal.pone.0029248 (PMC3237610; doi:10.1371/journal.pone.0029248)
Supplement: Table S1 — Clinical characteristics of 8 patients with AD-CMC from 3 families. (DOC) [file pone.0029248.s002.doc]

**Supplementary information submitted along with the following manuscript:**

**STAT1 hyperphosphorylation and defective IL12R/IL23R signaling underlie defective immunity in autosomal dominant chronic mucocutaneous candidiasis**

Sanne P. Smeekens^1,4,*^, Theo S. Plantinga^1,4,*^, Frank L. van de Veerdonk^1,4,*^, Bas Heinhuis^3^, Alexander Hoischen^2^, Leo A.B. Joosten^1,4^, Peter Arkwright^5^, Andrew Gennery^6^, Bart Jan Kullberg^1,4^, Joris A. Veltman^2^, Desa Lilic^6^ , Jos W.M. van der Meer^1,4,#^, Mihai G. Netea^#1,4,@^

**Supplemental Table 1. Clinical characteristics of 8 patients with AD-CMC from 3 families.**

|  | **Gender & age** | **Origin** | **Fungal infection** | **Hypo-thyroidism** | **other autoimmune disease** | **Eczema boils** | **Other diseases** | **Mutation identified by^2^** |
| --- | --- | --- | --- | --- | --- | --- | --- | --- |
| **Family #1** | | | | | | | | |
| Patient 1 * | Male, 67 y | NL | Oral, nails, skin | no | AI hepatitis | yes | Chest infections | NGS |
| Patient 2 * | Female 38 y | NL | Oral, nails, skin | no | AI hemolytic anemia Anti-phospholipid antibodies | no | Chest infections Bronchiectasis Pneumocystis pneumonia CMV Pulm. embolism | NGS |
| Patient 3 * | Male 37 y | NL | Oral, nails, skin | no | no | no | no | NGS |
| Patient 4 | Male  6y | NL | Oral, nails | no | no | no | no | Sanger |
| **Family #2** | | | | | | | | |
| Patient 1 | Female, 47 y | UK | Oral, nails, skin | yes | Irritable bowel syndrome | yes | Chest & sinus infections  Fe-deficiency anemia | Sanger |
| Patient 2 | Female 19 y | UK | Oral, nails, skin | yes | no | no | Fe-deficiency anemia | Sanger |
| Patient 3 | Male  2 y | UK | Oral, nails, perineum | No  Thyroid aabs | no |  | Chest infections  Fe-deficiency anemia | Sanger |
| **Family #3** | | | | | | | | |
| Patient 1 * | Female  40 y | UK | Oral  Nails  Skin  Vaginal | yes | no | yes | Chest  infections  Fe-deficient  anemia | Sanger |
| Patient 2 | Male  9 y | UK | Oral | yes | AI hepatitis | no | Mouth ulcers  Herpetic whitlow | Sanger |
| Patient 3 | Female  7 y | UK | Oral | yes | no | no | Mouth ulcers  Herpetic whitlow | Sanger |

*These patients have been previously reported in *van de Veerdonk et al., NEJM 2011.*
